# Relation between childhood trauma and social vulnerability in adulthood in patients with first episode psychosis

**DOI:** 10.1192/j.eurpsy.2025.2242

**Published:** 2025-08-26

**Authors:** P. Muñoz-Calero, P. Mola, A. Carrasco, W. Ahyed, M. D. Saiz, J. García-Albea, M. Díaz Marsá

**Affiliations:** 1 Instituto de Psiquiatría y Salud Mental, Hospital Clínico San Carlos, Madrid, Spain

## Abstract

**Introduction:**

The relationship between childhood adversity and psychosis has been the focus of extensive research in recent years. Studies suggest that individuals who experience significant adversity during childhood,such as abuse,neglect,or trauma, hava an increased risk of developing psychotic disorderser later in life.

Enviromental factor have been shown to play a signitficant role in the development of psychosis,often interacting with genetic predispositions.Nevertheless the relation between childhood trauma and social vulnerability in adulthood in patients with a first episode of psychosis (FEP) patients has not been studied.

**Objectives:**

The aim of this work is to study social factors in patients with childhood trauma and their impact on the development of a FEP.

**Methods:**

The sample was divided into 3 groups, controls,first episode psychosis patients with childhood trauma (FEP with CT) and first episode psychosis patients without childhood trauma (FEP without CT). 135 controls and 190 patients with FEP (58.42% with CT) were assesed through questionnaires on traumatic experiences, life stress events and a socio-demographic interviews. The likelihood of experiencing life stress events in the past year, social vulnerability,affective issues and substance use were examined using logistic regression models.

**Results:**

Four covariates demonstrated a significant association with the clinical group with CT: being without a partner (p < .01), unemployment (p < .01), a history of psychiatric conditions (p < .01), and migration status (p < .01). However, stressful events in adulthodd were not found to be significant.

**Conclusions:**

While childhood trauma does not seem to directly trigger re-traumatization in adulthood, it may contribute to place FEP patients in socially vulnerable circumstances that could lead to the development of psychotic symptoms.

**Disclosure of Interest:**

None Declared

